# An experimental drilling apparatus used for evaluating drilling risks related to natural gas hydrate

**DOI:** 10.1016/j.mex.2020.101019

**Published:** 2020-08-06

**Authors:** Tian-Jia Huang, Yu Zhang, Yi Wang, Xiao-Sen Li, Zhao-Yang Chen

**Affiliations:** a Key Laboratory of Gas Hydrate, Guangzhou Institute of Energy Conversion, Chinese Academy of Sciences, Guangzhou 510640, P. R China; b Guangdong Provincial Key Laboratory of New and Renewable Energy Research and Development, Chinese Academy of Sciences, Guangzhou 510640, P. R. China; c Guangzhou Center for Gas Hydrate Research, Chinese Academy of Sciences, Guangzhou 510640, P. R. China; d University of Chinese Academy of Science, Beijing 10083, P. R China

**Keywords:** Experimental drilling apparatus, Hydrate-bearing sediments, Drilling fluid, Hydrate dissociation, Wellbore stability

## Abstract

Some countries are trying to drill and exploit natural gas hydrate (NGH). However, the disturbance effects of drilling on the stability of NGH-bearing sediments are unclear. There are still few experimental apparatuses on this issue, and existing experimental apparatuses cannot comprehensively simulate the drilling process as well. In order to fill this gap in prior studies, an experimental drilling apparatus used for evaluating drilling risks related to NGH was developed. The apparatus consists of a high-pressure vessel with a drilling system, a drilling fluid injection system, a drilling fluid treatment system, and a data acquisition system. Hydrates can form in the high-pressure vessel placed inside a walk-in cold room. The drilling fluid can be cooled to the desired temperature by the drilling fluid treatment system and be injected into the drilling system by the drilling fluid injection system. The drilling system can simulate the comprehensive drilling process, including drilling feed, trip up & down operations, drilling fluid circulation, etc. 48 thermometers were inserted into the high-pressure vessel from the bottom. The thermometers uniformly distribute in the high-pressure vessel, and they could quickly and accurately measure the hydrate phase change process under high-pressure and low-temperature conditions.

• Simulate the drilling process in hydrate-bearing sediments.
• Evaluate the influence of drilling parameters (drilling fluid temperatures, drilling fluid circulation rates, etc.) on hydrate dissociation characteristics around the wellbore.
• Simultaneously evaluate the heat and mass transfer process in hydrate-bearing sediments during the drilling process.

## Graphical abstract

**Figure:**
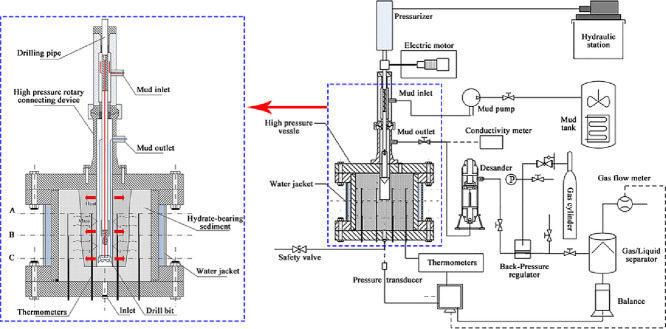

Specifications Table

**Table:** 

Subject Area	Engineering
More specific subject area	Exploitation of natural gas hydrate
Method name	An experimental apparatus for drilling in hydrate-bearing sediments
Name and reference of original method	
Resource availability	

## Method details

Natural gas has increasingly become a profitable alternative energy resource [1]. However, with the readily accessible reserves that can be found less and less, it is needed to obtain natural gas from conditions that are both more severe and more remote, such as deep ocean environments and permafrost environments [2]. Natural gas hydrate (NGH) has currently been recognized as a potential alternative to traditional energy resources. Many countries are trying to exploit NGH, and some exploitation projects have been carried out [3]. When drilling in hydrate-bearing sediments, the drilling may alter the temperature and pressure conditions of hydrate-bearing sediments and cause the NGH to dissociate into gas and water, inducing a decrease in sediment strength, which causes the wellbore collapse and open-hole enlargement [4]. Meanwhile, a large amount of methane gas (generated from the hydrate dissociation) inrushes into the drilling fluid and then may form hydrate again inside the pipes and valves of drilling equipment [5]. Till now, there is only limited literature on experimental tests about hydrate drilling risks. The bottleneck of this problem lies in the lack of a detection method and an experimental apparatus that can quickly and accurately measure the hydrate phase change under high-pressure and low-temperature conditions where NGH is suitable for existence. Furthermore, existing experimental apparatuses cannot comprehensively simulate the drilling process as well. Therefore, we developed a novel drilling experimental apparatus used for evaluating drilling risks related to NGH. The following is a detailed description of the experimental apparatus.

### Apparatus description

**Fig. 1:**
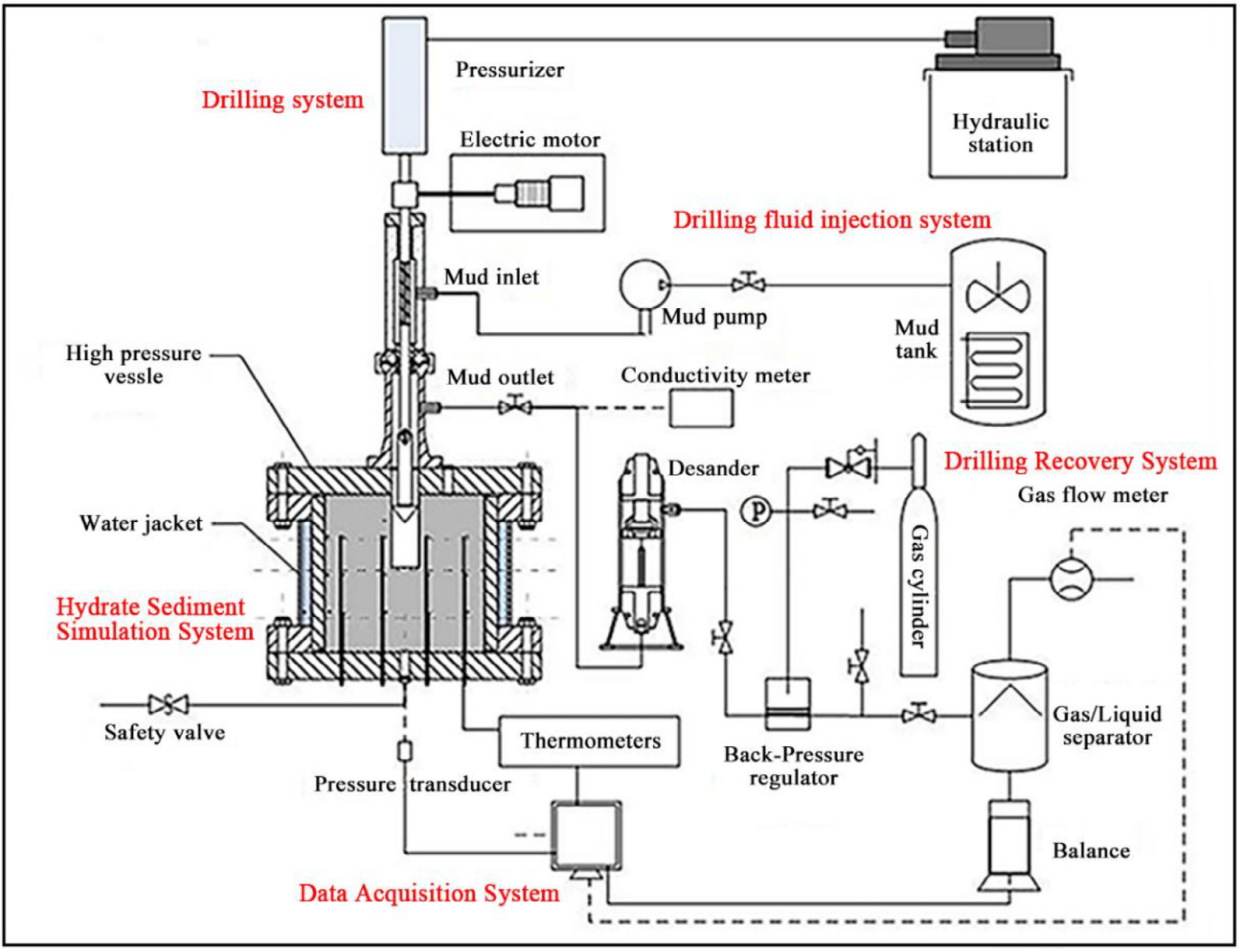
Schematic of the experimental apparatus.

Fig. 1 shows the schematic diagram of the experimental apparatus. The experimental apparatus consists of five parts, including a hydrate sediment simulation system, a drilling system, a drilling fluid injection system, a drilling fluid recovery system, and a data acquisition system. The operating pressure of the apparatus ranges from 0 to 30 MPa, and the operating temperature ranges from −5 to 20 °C.

**Fig. 2:**
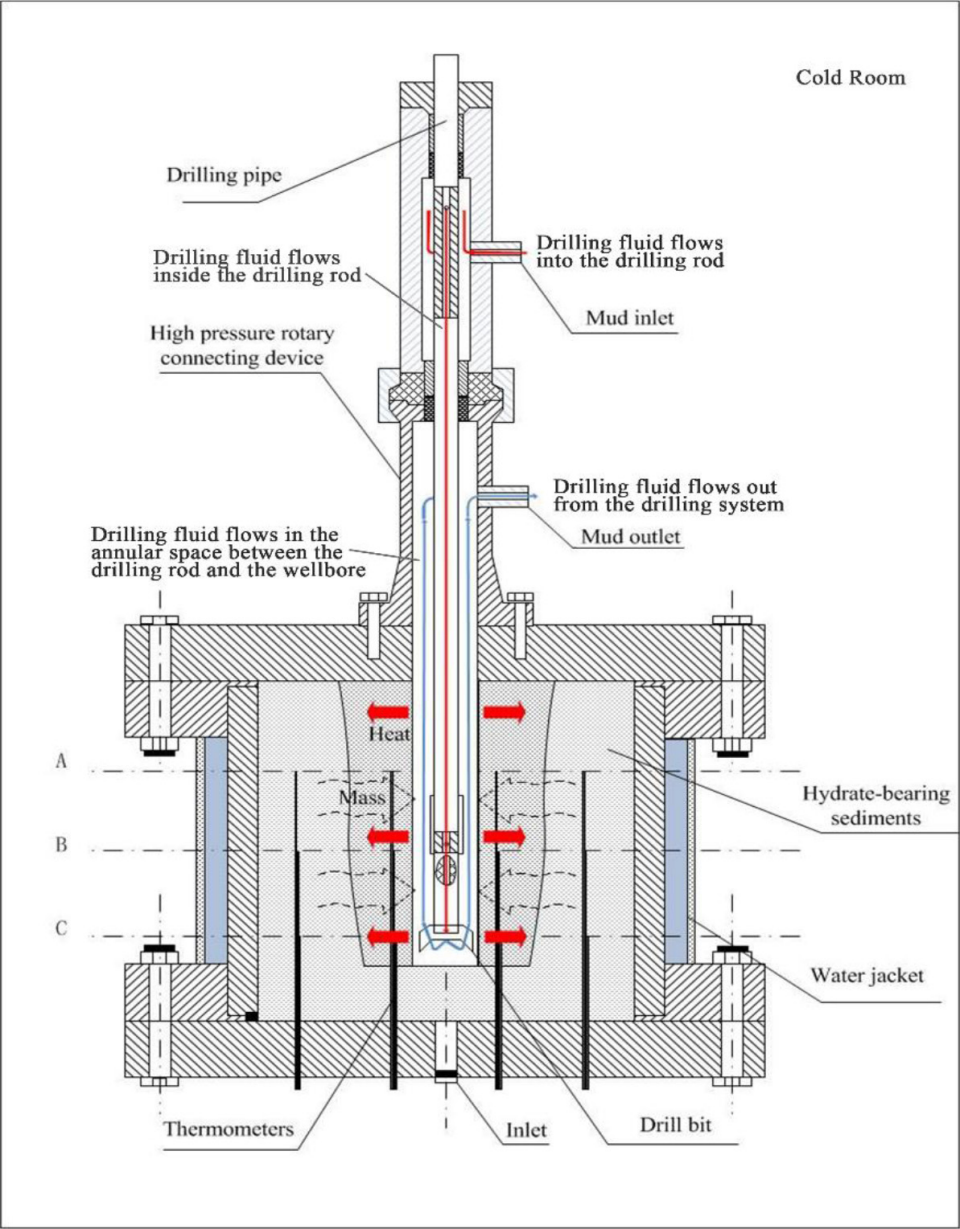
Schematic of the hydrate sediment simulation system.

**Fig. 3:**
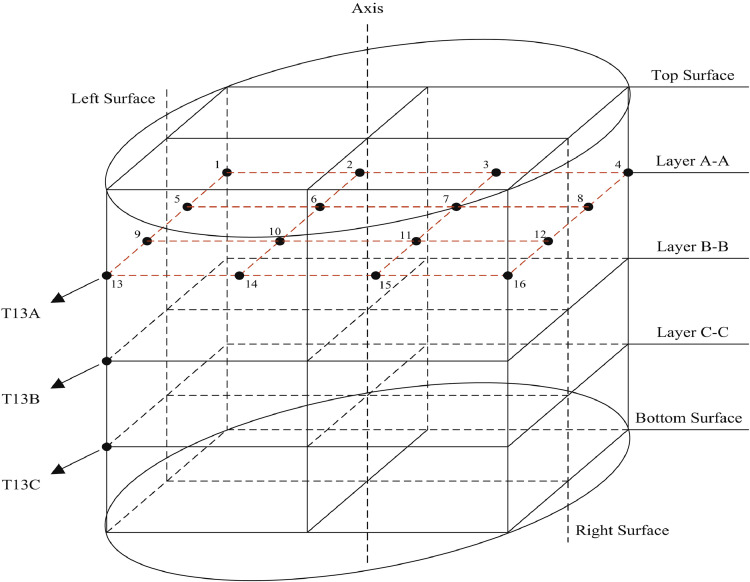
Schematic of the distribution of the thermometers in the vessel.

**Fig. 4:**
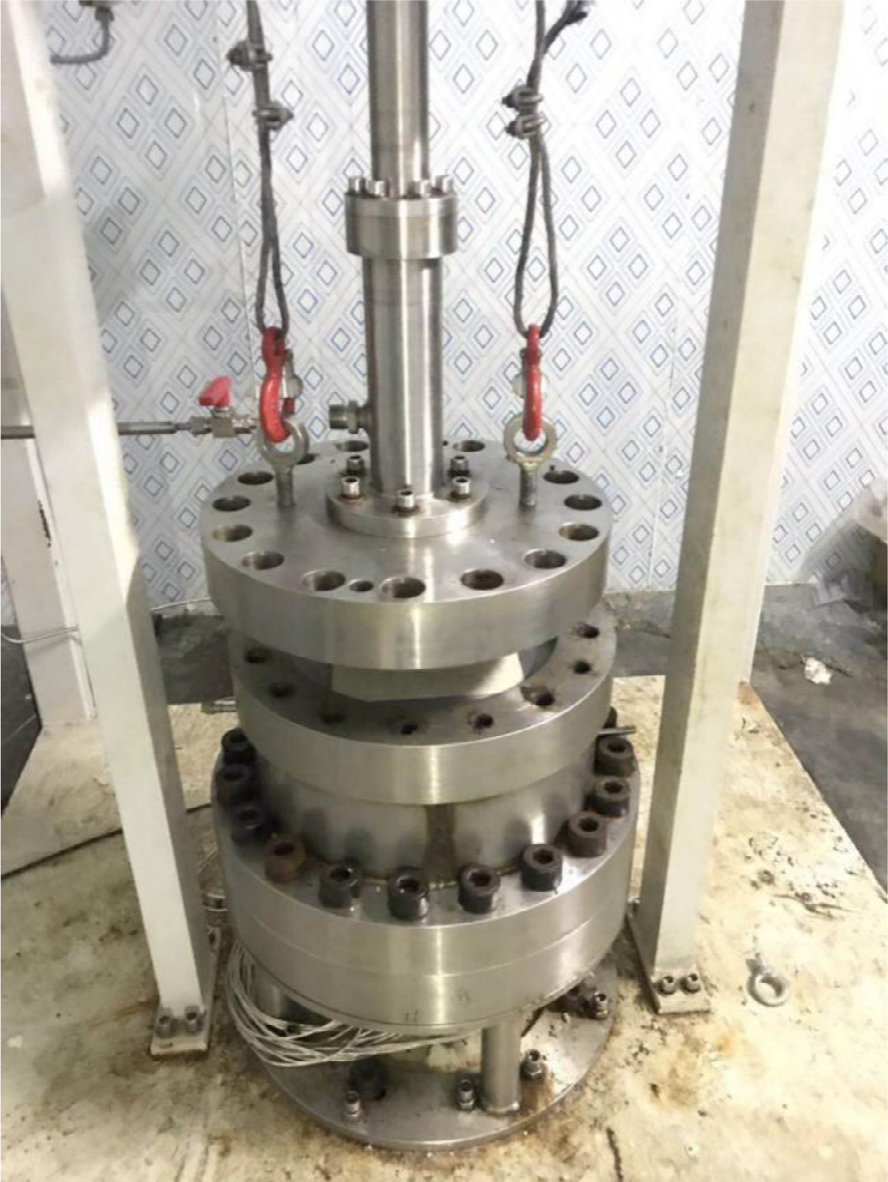
Picture of the high-pressure vessel.

The hydrate is formed in the hydrate sediment simulation system. As shown in Fig. 2, the hydrate sediment simulation system consists of a high-pressure vessel and an artificial core. The interior of the high-pressure vessel is cubic, and the side length and volume of the cubic are 180 mm and 5.8 L, respectively. The pressures at the center of the bottom of the high-pressure vessel, the drilling fluid inlet, and the drilling fluid outlet are measured by pressure transducers (TRAGAG NAT 8251.84.25171 0 – 40 MPa, ± 0.025%). Fig. 3 shows a schematic of the distribution layers of the thermometers (Pt100, −20 - 200 °C, ±0.1 °C), which are inserted into the high-pressure vessel from the bottom, in the artificial core. There are three horizontal layers named Layers A-A, B-B, and C—C inside the vessel, which divide the cubic vessel into 4 equal regions. Each layer (Layers A-A, B-B, and C—C) has 16 temperature measuring points. The high-pressure vessel with a water jacket is located in a cold-room so that hydrate-bearing sediments in the high-pressure vessel can be cooled to the desired temperature required for experiments. Fig. 4 shows a picture of the high-pressure vessel.

**Fig. 5:**
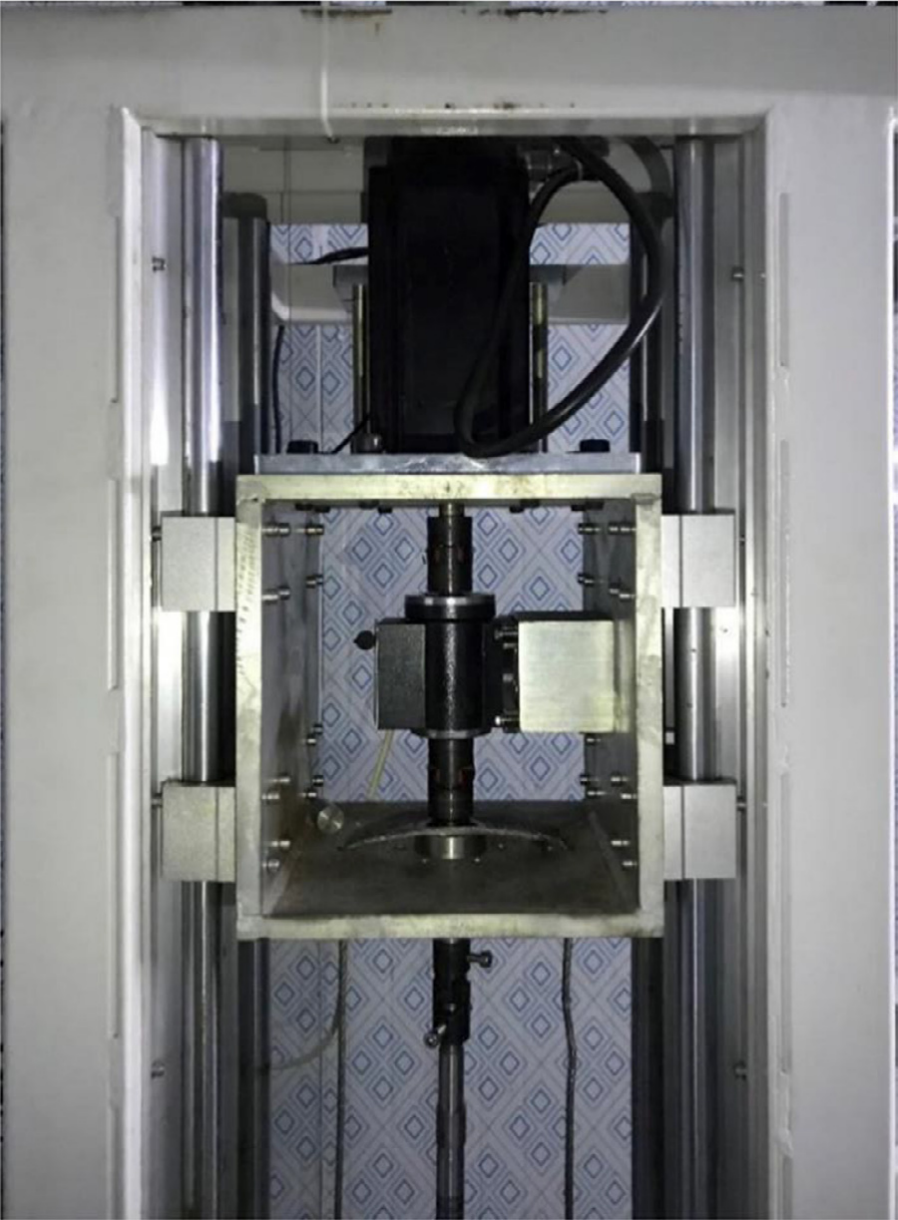
Picture of the drilling system.

**Fig. 6:**
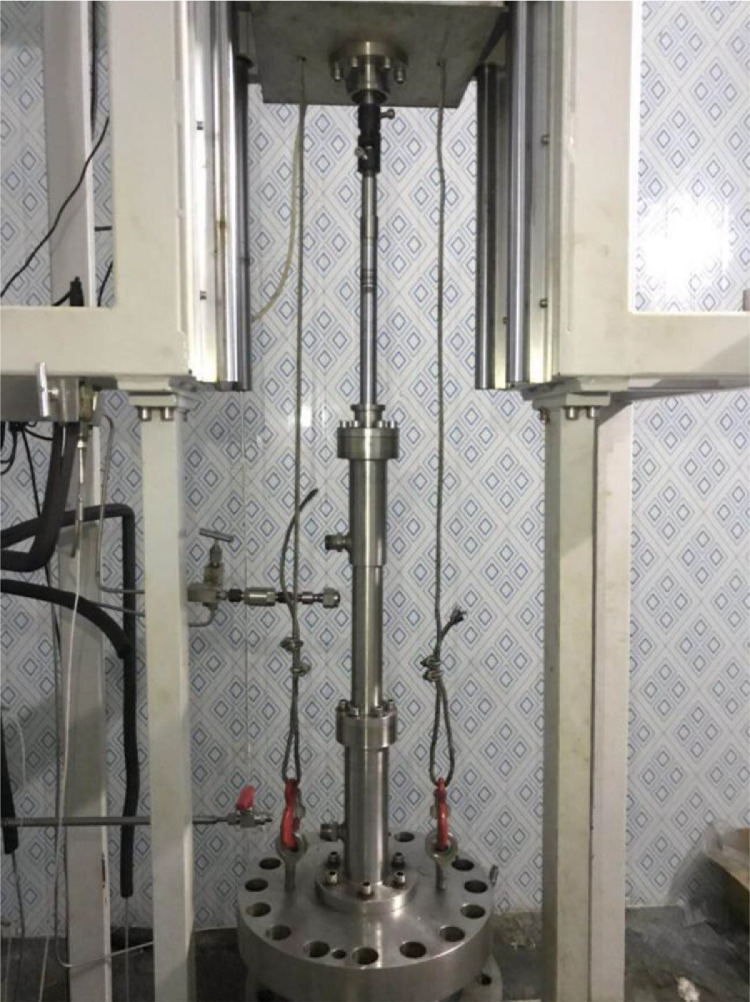
Picture of the connection between the drilling system and the high-pressure vessel.

The drilling system is used to simulate the drilling process. The drilling system consists of the support, a high-pressure rotary connecting device, two electric motors, and a drilling device. As shown in Figs. 1 and 2, one of the electric motors provides a necessary downward or upward force on the drill rod; another electric motor rotates the drill rod. The rotating speed of the drilling rod ranges from 0 to 200 r/min. The diameter of the drilling bit is 25 mm, and the maximum drilling depth is 180 mm. The drilling rod is hollow inside and equipped with a one-way valve. The torque of the drilling rod and the drilling depth can be measured by the torque sensor and the displacement sensor. The drilling system and the high-pressure vessel are connected by the high-pressure rotary connecting device through 6 screws so that the drilling bit can drill into hydrate-bearing sediments. Fig. 5 shows the picture of the drilling system, and Fig. 6 shows the picture of the connection between the drilling system and the high-pressure vessel.

The drilling fluid injection system, including a drilling fluid tank, a drilling fluid cooling unit, and a drilling fluid pump, is used to provide the drilling fluid within a temperature range of 0 - 20 °C and a flow range of 0 - 1 L/min. The drilling fluid tank is provided with a cooling unit and a stirring unit. The drilling fluid with the desired temperature and flow can be injected into the drilling rod from the drilling fluid tank via the drilling pump; then the drilling fluid flows out from the drilling bit through a one-way valve, and finally flows out from the mud outlet via the annular space between the drilling rod and the wellbore, as shown in Fig. 2.

The drilling fluid flowing out from the drilling system can be gathered through the drilling recovery system. The drilling fluid recovery system consists of a high-pressure desander, a backpressure regulator, a gas-liquid separator, a gas flowmeter, a balance, and a conductivity meter. The high-pressure desander can reduce the sand content in the drilling fluid flowing out from the drilling fluid outlet. The backpressure regulator, connected to the outlet of the high-pressure desander, is used to control the pressure of the high-pressure vessel. The balance and gas flowmeter are used to measure the mass of liquid and gas produced from the vessel, respectively. Besides, the conductivity meter (CON3000, 0.001 µS/cm~0.1 mS/cm) can measure the conductivity of the drilling fluid flowing out from the drilling fluid outlet.

The data acquisition system includes the matching software and several sensors. The data measured by each sensor, including temperature, pressure, drilling depth, drilling torque, total quality of the cumulative liquid and gas produced from the vessel, and conductivity of drilling fluid flowing out from the outlet, can be recorded and saved at a regular interval by the matching software.

### Experiments description

The experiment procedure essentially comprises two stages: a) sample preparation; b) drilling in hydrate-bearing sediments.

#### Sample preparation

Since it is hard to recover core samples from actual NGH-bearing sediments and maintain their intact status, artificial cores are designed to simulate the porous nature of NGH reservoirs. There are two kinds of artificial cores in experiments. One is the unconsolidated core, which has high permeability but low mechanical strength. Another is the consolidated core made by mixing quartz sands and epoxy resin. The consolidated core has high mechanical strength, and its permeability can be controlled.

The artificial core is tightly packed into the high-pressure vessel. After the artificial core is prepared, the gas or saline solution is injected into the high-pressure vessel by a booster pump until the pressure in the high-pressure vessel reaches a desired value. Meantime, the temperature of the cold room is set to the desired experimental temperature. Subsequently, hydrates will form gradually in the artificial core.

Take the cases in this study for example, for the unconsolidated sediments, quartz sands with particle sizes from 40 – 70 mesh number (0.425mm-0.212 mm) without any additive were tightly packed into the high-pressure vessel. The weight of the quartz sands packed into the vessel is approximately 8.2 kg. For the consolidated sediments, the quartz sands with particle sizes from 40 – 70 mesh number (0.425mm-0.212 mm) were firstly mixed thoroughly with epoxy resin and then packed into the high-pressure vessel. The mass ratio of epoxy resin to quartz sands used in experiments is 1:10. In the experiments, the weight of the cementation packed into the high-pressure vessel is approximately 10.0 kg.

#### Drilling in hydrate-bearing sediments

After hydrate-bearing sediments are prepared, the drilling experiments are carried out following these steps:

1) Turn on the drilling fluid cooling unit, the drilling fluid in the mud tank is cooled to the desired temperature;
2) Adjust the set pressure value of the back-pressure regulator to the pressure required by experiments, and control the pressure inside the high-pressure vessel to reach the experimental pressure;
3) Set the values of drilling fluid flow, drill rod rotation speed, and drill rod movement speed;
4) Inject the drilling fluid in the drilling fluid tank into the drilling system by the mud pump at a constant rate;
5) At the same time, turn on the electric motors, making the drill rod to move downward at a set speed and rotate at a set rotating speed;
6) When the drill bit reaches its maximum drilling depth, the drill rod stops moving downward and continues to rotate until the experiment is ended;
7) The data required are recorded at certain time intervals during the experiment process by the software;
8) At the end of the experiments, the high-pressure vessel can be opened to observe the condition of hydrate-bearing sediments directly.

## Some method results

Tetra-n‑butyl ammonium (TBAB) hydrate was formed in the unconsolidated or consolidated cores to simulate hydrate-bearing sediments because TBAB hydrate can form at atmospheric pressure and near room temperature and has some similar physical and structural properties with NGH. Therefore, it is a practical and easier method to carry out drilling experiments in TBAB hydrate-bearing sediments.

The phase equilibrium condition of TBAB hydrate is decided by the TBAB solution concentration. Normally two types of TBAB hydrate can grow simultaneously in aqueous solution, type A and type B [6]. The type A TBAB hydrate has a hydration number of 26.0, and a 40 wt% congruent melting point of 12 °C [6]. 40 wt% TBAB solution was selected to form TBAB hydrate in the experiments. According to the previous study, the TBAB solution with a concentration of 40 wt% can totally form type A TBAB hydrate [7].

### Formation of TBAB hydrate

**Fig. 7:**
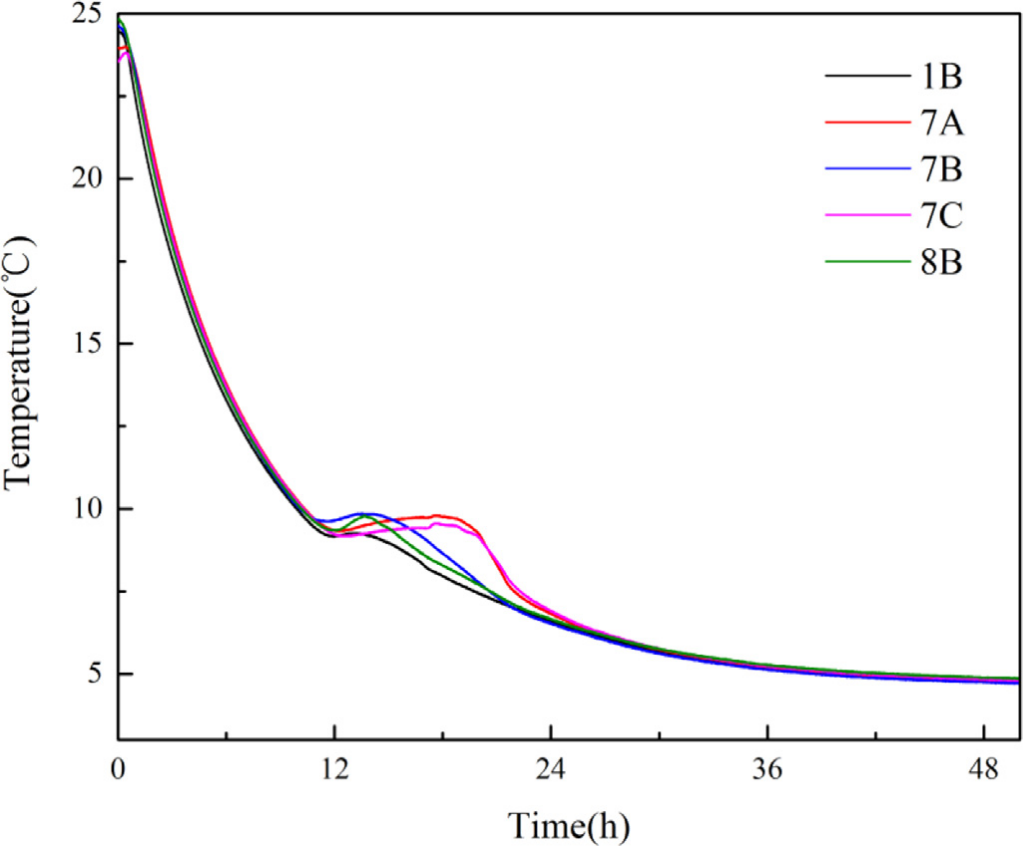
Temperature changes at different measurement points during the TBAB hydrate formation process [8].

Fig. 7 shows the temperatures of measurement points 1B, 7A, 7B, 7C, and 8B change with time during the TBAB hydrate formation process. Typically, the temperature of the TBAB solution firstly fallen below the phase equilibrium temperature due to cooling. No hydrate formed during this process. Then, the temperature of the TBAB solution rose slightly because hydrate nucleation is an exothermic process. Finally, large hydrate crystals began to grow based on core particles, and the temperature gradually decreases to the air bath temperature because of the heat transfer. Note that the duration of the nucleation process of different points is different due to the different core structure of each position.

### Effects of drilling fluid temperature

**Fig. 8:**
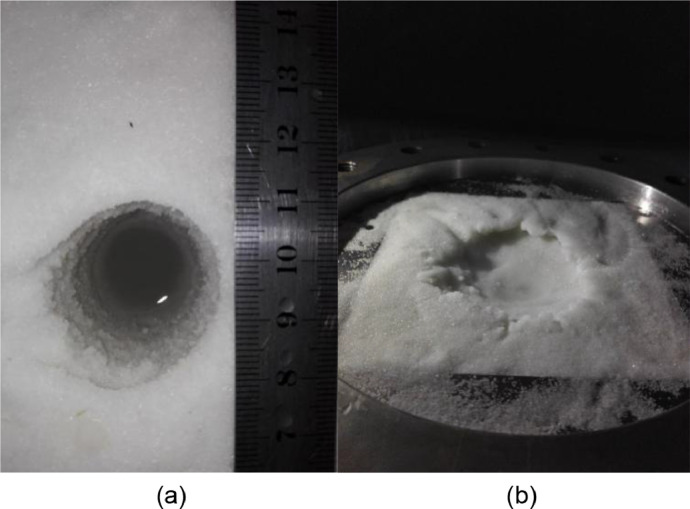
Photos of the wellbore after drilling of experiments 1 and 2. (a: the photo after drilling with the drilling fluid temperature of 4 °C; b: the photo after drilling with the drilling fluid temperature of 8 °C).

The TBAB solution was injected into the unconsolidated core in the high-pressure vessel to form the TBAB hydrate-bearing sediments at 4 °C, and the temperatures of drilling fluid are 4 °C and 8 °C for experiments 1 and 2, respectively. Fig. 8 shows the photos of the wellbore after experiments 1 and 2. It can be found that the wellbore kept steady after the sediment breaking process when the drilling fluid temperature is 4 °C, while the wellbore collapsed significantly when the drilling fluid temperature is 8 °C. It illustrates that the temperature of the drilling fluid significantly affects the stability of the wellbore during the drilling process.

**Fig. 9:**
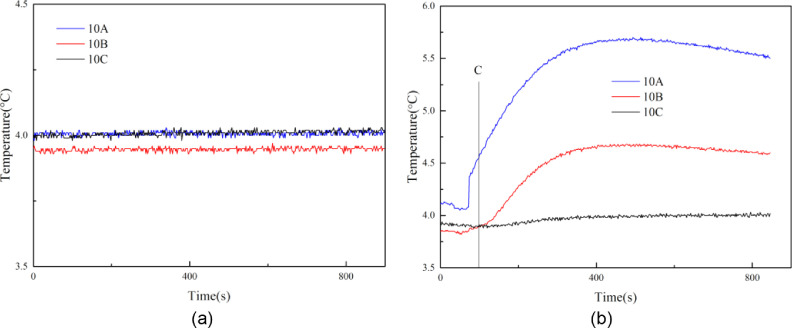
(a)Temperatures change with time at different measurement points in experiment 1; (b) Temperatures change with time at different measurement points in experiment 2.

Fig. 9 gives the temperatures change at different measurement points during experiments 1 and 2. When the drilling fluid temperature is 4 °C, the temperatures at measurement points 10A, 10B, and 10C change little during the whole experiment. It indicates that the hydrate is in a stable condition during the drilling process. However, when the drilling fluid temperature is 8 °C, the temperatures at measurement points 10A, 10B, and 10C increase gradually after the end of the sediment breaking process (Point C in Fig. 9(b)). In such a case, the hydrate around the wellbore dissociates gradually because of heat transfer. Therefore, the wellbore collapsed totally at the end of experiment 2, as shown in Fig. 8.

**Fig. 10:**
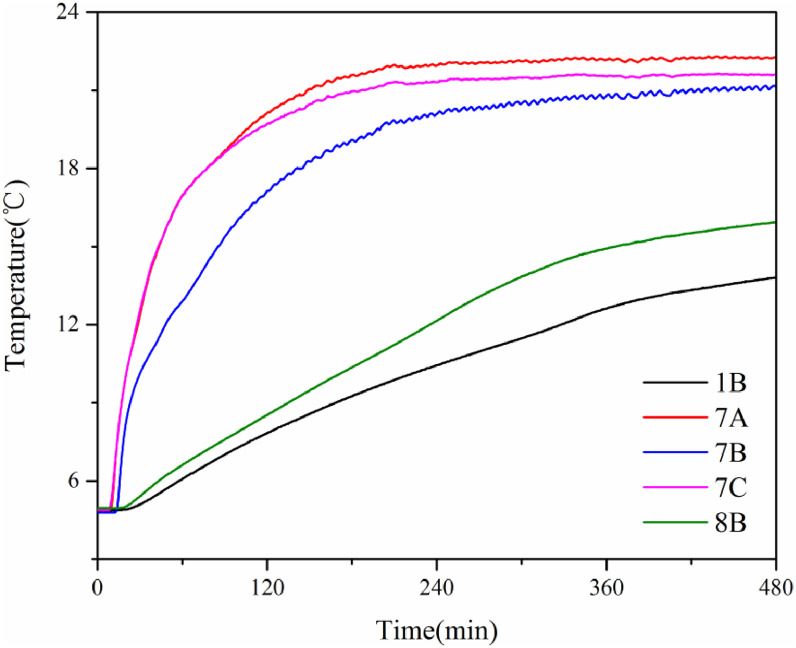
The temperatures of measurement points 1A, 7A, 7B, 7C, and 8B change with time in experiment 3 [8].

Fig. 10 shows the temperatures of measurement points 1B, 7A, 7B, 7C, and 8B change with time in experiment 3, which was performed in consolidated sediment with the drilling fluid temperature of 22 °C. Since the drilling fluid temperature is higher than the initial temperature of the sediment, the temperature of the sediment around the wellbore gradually increases with the experiment beginning. However, the temperature rise rate of each measurement point is significantly different. Therefore, the temperature gradually decreases with the distance from the wellbore increasing. Finally, the temperatures of measurement points 7A, 7B, and 7C reached a stable value that is near the drilling fluid temperature, while the temperature of measurement points 1B and 8B did not reach a stable value at the end of the experiment.

**Fig. 11:**
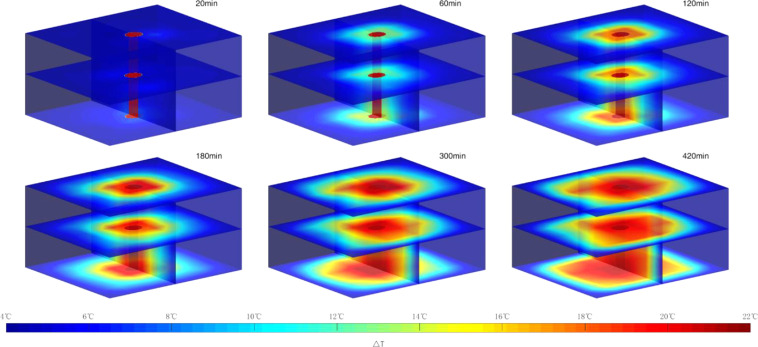
Spatial distributions of temperature in sediments at different time in experiment 3.

Fig. 11 shows the three-dimensional temperature distributions in the high-pressure vessel for experiment 3 at different times. The red cylinder in the middle represents the wellbore. Hydrate dissociation is an endothermic process in which heat must be supplied to break the hydrogen bonds [9]. As shown in Fig. 11, the temperature of the sediment gradually increases with the high-temperature drilling fluid circulating, which could cause the hydrate near the wellbore gradually dissociates. At the 420th min of experiment 3, the temperature in most areas in the high-pressure vessel is higher than 12 °C, which is harmful to drilling safety.

In summary, the drilling apparatus in this study can experimentally simulate the dynamic drilling process in hydrate-bearing sediments with different drilling fluid properties, rod rotation speeds, and rod movement speeds. The temperature distribution around the wellbore, the pressure change in the high-pressure vessel, the water production, the gas production, and the conductivity change of drilling fluid during the drilling process can be obtained by the apparatus. Therefore, this experimental drilling apparatus can provide an analogy between drilling operations in a field and drilling experiments in an indoor laboratory, and investigate the hydrate dissociation characteristics around the wellbore and wellbore stability during the drilling process.

## Declaration of Competing Interest

The authors declare that they have no known competing financial interests or personal relationships that could have appeared to influence the work reported in this paper.
